# Redox Conversions of 5-Methyl-6-nitro-7-oxo-4,7-dihydro-1,2,4triazolo[1,5-a]pyrimidinide L-Arginine Monohydrate as a Promising Antiviral Drug

**DOI:** 10.3390/molecules26165087

**Published:** 2021-08-22

**Authors:** Alexandra Ivoilova, Ludmila V. Mikhalchenko, Anton Tsmokalyuk, Marina Leonova, Andrey Lalov, Polina Mozharovskaia, Alisa N. Kozitsina, Alla V. Ivanova, Vladimir L. Rusinov

**Affiliations:** 1 Institute of Chemical Technology, Ural Federal University, 19, Mira St, 620002 Ekaterinburg, Russia; shyra-8@mail.ru (A.I.); atsmok@mail.ru (A.T.); mpnrevda@gmail.com (P.M.); a.n.kozitsina@urfu.ru (A.N.K.); a.v.ivanova@urfu.ru (A.V.I.); 2 Zelinsky Institute of Organic Chemistry, Russian Academy of Sciences, 47, Leninsky Prospect, 119991 Moscow, Russia; mlv@ioc.ac.ru (L.V.M.); marina1235052@gmail.com (M.L.); land@ioc.ac.ru (A.L.); 3 Institute of Organic Synthesis, Ural Branch, Russian Academy of Sciences, 22 Sofia Kovalevsky St, 620137 Ekaterinburg, Russia

**Keywords:** nitro-1,2,4-triazolo[1,5a]pyrimidines, Triazid, nitro group transformations, nitroaromatic compounds, nitroheterocyclic compounds, antiviral drugs, cyclic voltammetry, ESR spectroscopy

## Abstract

This article presents the results of a study of electrochemical transformations in aqueous and aprotic media of 5-methyl-6-nitro-7-oxo-4,7-dihydro-1,2,4-triazolo[1,5-a]pyrimidinide l-arginine monohydrate (**1a**, Triazid) obtained by electrochemical methods and ESR spectroscopy. The effect of pH on the current and the reduction potential of **1a** in an aqueous Britton–Robinson buffer solution was studied. It was found that 1a is irreversibly reduced in aqueous acidic media on a glassy carbon electrode in one stage with the participation of six electrons and the formation of 5-methyl-6-amino-7-oxo-1,2,4-triazolo[1,5-a]pyrimidin. The electroreduction of **1a** in DMF on a background of tetrabutylammonium salts proceeds in two stages, controlled by the kinetics of second-order reactions. In the first stage, the reduction of **1a** is accompanied by protonation by the initial compound of the basic intermediate products formed in the electrode reaction (self-protonation mechanism). The second quasi-reversible stage of the electroreduction **1a** corresponds to the formation of a dianion radical upon the reduction of the heterocyclic anion 5-methyl-6-nitro-7-oxo-4,7-dihydro-1,2,4-triazolo[1,5-a]pyrimidin, which is formed upon the potentials of the first peak. The ESR spectrum of the radical dianion was recorded upon electroreduction of Triazid in the presence of Bu_4_NOH. The effect of the formation of ion pairs on the reversibility of the second peak of the **1a** transformation is shown. A change in the rate and regioselectivity of the protonation of the dianion radical in the presence of Na^+^ and Li^+^ ions is assumed. The results of studying the electroreduction of **1a** by ESR spectroscopy with a TEMPO trap make it possible to assume the simultaneous formation of both a nitroxyl radical and a radical with the spin density localized on the nitrogen at the 4 position of the six-membered ring.

## 1. Introduction

It is known that influenza viruses, because of their mutation, are one of the most common and widely spread illnesses. This leads to some difficulties in dealing with influenza. In this regard, the creation of new, more active, and safe medicinal antiviral drugs does not lose its relevance [1,2,3,4].

Currently, there is a certain pharmacological niche of drugs based on nitro compounds that are effective against various types of influenza. In recent decades, nitro compounds of an aromatic and heterocyclic nature have attracted considerable attention since they are widely used throughout the world as antimicrobial, antiviral, antiparasitic, and radiosensitizing agents [5,6]. The mechanism of action of nitroheterocyclic therapeutic agents is currently not completely understood. It is known that medicinal substances, especially nitro compounds, undergo redox transformations in the human body. Some authors have described the connection between process reduction of the nitro group and the biological, antiviral action of these compounds [6,7]. Therefore, the research and development of models that can describe the redox transformation of nitro compounds is an important task. The solutions found through this aim will help advance the understanding of the biological action of pharmaceutical preparations in living organisms based on nitro compounds.

In a wide range of synthetic heterocyclic compounds with antiviral action, a significant place is occupied by azoloazines containing a bridging nitrogen atom in the structure of the molecule 1,2,4-triazolo [1,5-a]triazines and 1,2,4-triazolo[1,5-a]pyrimidine. The interest in azoloazines has resulted from the structural similarity they have to nucleic bases [8]. On the basis of nitro-containing azoloazinium compounds, researchers of the Ural Federal University, named after the first President of Russia, B.N. Yeltsin; the I.Ya. Postovsky Institute of Organic Synthesis of the Ural Branch of the Russian Academy of Sciences (IOS UB RAS); and the A.A. Smorodintsev Research Institute of Influenza of the Ministry of Health of Russia have developed a new class of substances—potential drugs with a wide range of antiviral activity [9]. Triazavirin^®^, or riamilovir (sodium salt of 2-methylthio-6-nitro-7-oxo-1,2,4-triazolo[5,1-c][1,2,4]triazine, dihydrate), is the first drug based on this class of compounds. In addition, this drug is included in the register of medicines of the Russian Federation. In the course of its clinical use, Triazavirin has been used in the etiotropic therapy of influenza, acute respiratory viral infections [10,11], and tick-borne encephalitis [12]. Triazavirin contributes to a reduction in the duration of the main symptoms of these diseases and a significant decrease in the level of the re-isolation of viruses. The drug has been successfully used in COVID-19 hospitals as an etiotropic agent in the treatment of coronavirus infection, as well as a means of drug prevention [13,14].

There are a number of structural analogs of Triazavirin^®^. The most promising compound, as a molecule with higher antiviral activity against various strains of the virus in in vivo systems, is Triazid (5-methyl-6-nitro-7-oxo-4,7-dihydro-1,2,4triazolo[1,5-a]pyrimidinide monohydrate) [15,16]. Triazid has passed the first phase of clinical trials, which showed safety, good tolerance, and rapid absorption of the drug [15]. All these results indicate that Triazid is a promising drug to promote on the pharmaceutical market. In this regard, the question of studying its redox transformations is currently of interest.

The electrochemical behavior of monoheterocyclic nitro compounds, for example, pyridine, pyrazole, or imidazole derivatives, is most often similar to the behavior of aromatic nitro compounds [17]. Apparently, the aromaticity of the system is markedly reduced in a number of condensed heterocyclic structures with a bridging nitrogen atom [18]. In addition, new reaction centers, which have the ability to accept electrons and protons, appear. The reciprocal influence of these centers can be so strong that it can cause a radical change in the reactivity of particles [19]. This is probably why the results of studying the reactivity of particles formed from condensed heterocyclic compounds in electrochemical reactions cannot always fit into the framework of conventional concepts.

We previously studied the reduction of the active substance of Triazavirin^®^ and its derivatives (2-R-6-X-7-oxo-1,2,4-triazolo[5,1-c][1,2,4]triazin) in aqueous solutions and aprotic media by electrochemical methods and ESR spectroscopy [20]. Possible mechanisms of its transformation were proposed, and the obtained results indicated a radical mechanism of electroreduction (ER). Previously, we also showed that the reduction of Triazid occurs with the participation of the nitro group of the drug conjugated with the heterocyclic system. At the same time, possible intermediate and final reaction products of the Triazid compound were not studied, because we considered the electroreduction of the nitro group of Triazid only from the point of view of the analytical determination of its maintenance in aqueous solutions [21]. Therefore, the goal of this work was to study the possible pathways of the redox transformation of the molecule 5-methyl-6-nitro-7-oxo-4,7-dihydro-1,2,4-triazolo[1,5-a]pyrimidinide l-arginine monohydrate (Triazid), the comparison of its reactivity with the sodium salt of 2-methylthio-6-nitro-7-oxo-1,2,4-triazolo[5,1-c][1,2,4]triazin, dihydrate (Triazavirin^®^), and the identification of possible intermediate products of the electroreduction of Triazid using electrochemical methods and ESR spectroscopy.

## 2. Experimental Section

### 2.1. Materials and Reagents

**Scheme 1:**
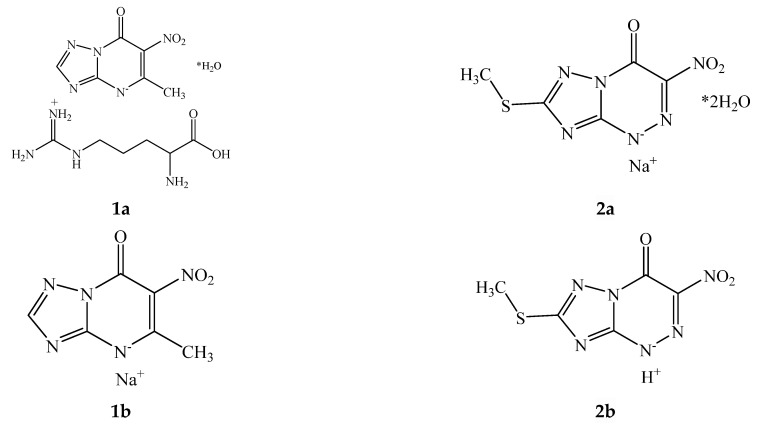
Structural formulas of compounds **1a**, **1b**, **2a**, **2b**.

5-Methyl-6-nitro-7-oxo-4,7-dihydro-1,2,4-triazolo[1,5-a]pyrimidinide l-arginine monohydrate (**1a**, Triazid), sodium salt of 5-methyl-6-nitro-7-oxo-4,7-dihydro-1,2,4-triazolo[1,5-a]pyrimidine (1b), sodium salt of 2-methylthio-6-nitro-7-oxo-1,2,4-triazolo-[5,1-c][1,2,4]triazin, dihydrate (**2a**, Triazavirin^®^), and 2-methylthio-6-nitro-7-oxo-1,2,4-triazolo-[5,1-c][1,2,4]triazin (**2b**) were synthesized at the Department of Organic and Biomolecular Chemistry, Ural Federal University. The structure of the compounds (Scheme 1) was determined using NMR spectroscopy, IR and UV spectroscopy, and elemental analysis [22,23,24].

To conduct a study of the electroreduction of substances, aqueous solutions of 0.1 M nitric acid of the puriss grade from the USA manufacturer Sigma-Aldrich^®^, St. Louis and Britton–Robinson (BR) buffers were used. The solutions were prepared in distilled water. Britton–Robinson solutions were prepared by mixing orthophosphoric, acetic, and boric acids and adjusting to the required pH with sodium hydroxide according to [25]. We used acids and salts of the puriss grade from the Spain Barcelona manufacturer PanReac without additional purification. To prepare solutions, we used deionized water obtained on a DVS-M/1NA (18)-N unit from Mediana-Filter, Moscow,Russia.

To carry out the study in aprotic solutions, acetonitrile of the puriss. spec. grade from the USA manufacturer PanReac was used without additional purification. In addition, DMF and DMSO of the puriss. spec. grade from the USA manufacturer Sigma-Aldrich^®^ with preliminary distillation [26] in the presence of a molecular sieve were used. We used lithium perchlorate and tetrabutylammonium tetrafluoroborate of the puriss. spec. grade from the USA manufacturer Sigma-Aldrich^®^.

To detect the radical, *N*-(1-hydroxy-2,2,6,6-tetramethylpiperidin-4-yl-)-2-methylpropanamide hydrochloride (TMTH) (Novosibirsk Institute of Organic Chemistry, Russia) was used as a spin probe [27,28]. As a spin trap, 3,3,5,5-tetramethyl-1-pyrroline N-oxide (TEMPO) (Sigma-Aldrich^®^, St. Louis, MI, USA, CAS No. 244007) was used [29,30].

### 2.2. Electrochemical Devices and Methods

Cyclic voltammograms (CVs) and chronoamperograms (CAs) were carried out using a μAutolab Type III potentiostat/galvanostat (Metrohm, Switzerland). Glassy carbon disks (Ø 2 and 5 mm stationary or rotating, respectively) were used as a working electrode. The surface of the working electrode was mechanically cleaned before each measurement. A saturated Ag/AgCl/KClsat electrode acted as a reference electrode in an aqueous medium, and a graphite electrode was used as the auxiliary electrode (Metrohm, Switzerland). The potentials of the working electrode in aprotic solutions were measured relative to a silver chloride reference electrode with two Ag/AgCl/KClsat/DMF membranes or a saturated calomel electrode (SCE). The CA was recorded at the potentials of the limiting current of electroreduction of compounds in the time interval of 0 < t < 5 s, and the logarithmic analysis of the chronoamperograms was performed in the interval of 1 < t < 2 s. The working solutions were purged with argon with a purity of 99.9% for 15 min before each measurement. Aqueous buffer solutions of Britton–Robinson (BR) or 0.1 M nitric acid were used as supporting electrolytes. The pH measurements were carried out on an Expert-pH ion meter (Econiks-Expert, Russia) and an EV-74 universal ion meter.

Each voltammogram was recorded three times. The mean peak current and the confidence interval were calculated (*p* = 0.95.)

### 2.3. ESR Spectroscopy with Preliminary Electrochemical Generation

ESR spectra were registered using a Bruker Elexsys E 500 spectrometer (Germany, Rheinstetten) with an ER4122SHQE resonator and Bruker EMX 6/1 (Germany Rheinstetten) spectrometer equipped with ER4102ST rectangular cavity equipped with two electrode convex Pt/Pt electrolytic cells controlled by a Zahner IM-6 potentiostat.

The generation of Triazid reduction products with a concentration of 0.01 M was carried out with the addition of a 0.01 M spin probe TMTH over 1/5/10/15 min. After the indicated time intervals, an aliquot of the solution was taken, and the ESR spectrum was recorded. To control the number of probes appearing in the background solution, the products were generated in a similar way and at the same potential without adding Triazid. Aliquots were taken after 1/5/10/15 min of generation, and the ESR spectra were recorded.

Before each experiment, the working solution was purged with argon.

Simultaneous Electrochemical Experiment ESR (SEESR) was used to detect paramagnetic particles during **1a** reduction. The spectra of the radical anion were recorded at a potential of −2 V in a two-electrode Pt/Pt electrochemical cell placed in the resonator of an ESR spectrometer. The half-life of the species detected was less than 0.01 s.

All quantum chemical calculations in the field of ESR were performed by the ORCA [31,32,33] ver. 5.0.0 program package using a hybrid PBE0 [34] density functional in a triple-zeta basis set with two polarization functions def2-TZVPP [35] and atom-pairwise dispersion correction with the Becke–Johnson damping scheme (D3BJ) [36], and respect to the effects of solvation in the SMD continuum [37] were included in all calculations. TightSCF convergence criteria were applied throughout. Full geometry optimization with TightOpt convergence criteria was carried out to find stationary points on the potential energy surfaces. Numerical harmonic frequency calculations were used to obtain thermodynamic quantities and verify that all stationary points found were local minima. Visualization of molecular orbitals were produced using the Avogadro program [38,39].

ESR spectra were recorded with a TEMPO spin trap [29,30] after preliminary generation of Triazid **1a** reduction products with a concentration of 0.1 M in DMSO in a conventional electrochemical cell for 15 min in the presence of a spin trap. An aliquot of the resulting solution was taken, and the ESR spectrum was recorded.

The simulation and visualization of the calculated ESR spectra with traps were performed using the EasySpin 5.3 software package [40].

## 3. Results and Discussion

### 3.1. Electrochemical Behavior of Compounds **1a** and **1b** in Aqueous Media

**Figure 1:**
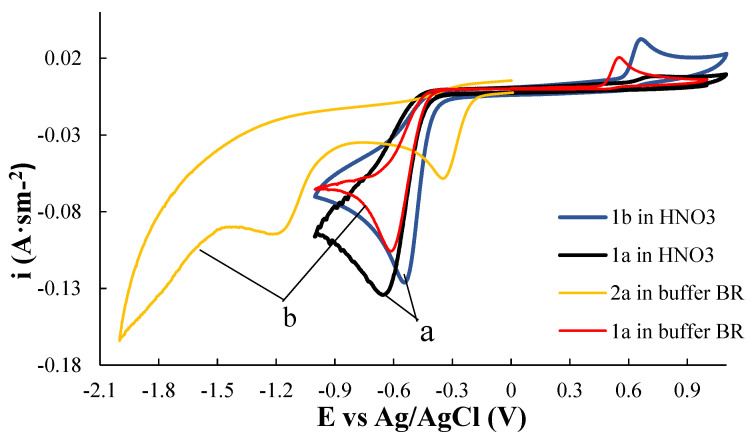
CVs of 5 mM compounds **1a** and **1b** in 0.1 M HNO_3_ (a) and (2a) and (1a) in a BR buffer solution (pH 2) (b) using glassy carbon electrode (GCE). *V* = 50 mVs^−1^. Potentials were measured relative to Ag/AgCl/KCl_sat_.

Figure 1 shows the CV of the first stages of the electroreduction of the studied compounds in an aqueous acidic medium.

The investigated compounds **1a** and **1b** in 0.1 M HNO_3_ were irreversibly reduced at the potential values –0.71 and –0.56 V, respectively. These potentials are comparable to the potentials of heterocyclic nitro compounds with a nitro group in their structure [6,17,19,41,42]. The value of the potential of the electroreduction of **1a** in a buffer solution at a pH of 2 is −0.63 V. Previously, we studied the electroreduction of compound **1a** in a buffer solution with a pH value of 2 [20]. A comparison of the CV of compounds **1a** and **2a** showed that under the same conditions (BR pH = 2), the latter undergoes reduction processes at potential values 250 mV higher than the former. The difference in the ER potentials is probably due to the presence of an additional electronegative nitrogen atom in the six-membered ring of the condensed heterocyclic system **2a**. Another difference in the electrochemical behavior of these nitro compounds is the fact that in acidic and neutral media, **2a** is reduced in two subsequent, irreversible stages, while the irreversible peak of the ER of **1a** is the only one up to the background discharge potential (Figure S1 Supplementary Materials).

As shown in Figure 1, irreversible peaks of the ER products **1a** and **1b** are observed in nitric acid at potentials +0.68 and +0.67 V, respectively, on the anodic part of the voltammograms after changing the direction of the potential sweep. The anion of the corresponding hydroxylamino derivative is probably oxidized at these potentials. The closeness of the values of these oxidation potentials indicates the formation of electroreduction products similar in structure to compounds **1a** and **1b**.

To determine the effective number of electrons n_e_ involved in the electrochemical reduction reaction, the current of the first stage of the reduction of compound **1a** at a pH of 2 in the CV was compared with the current calculated using the Rendles–Shevchik equation for irreversible electrochemical reactions [43] and the current of the standard reversible redox system Fe(CN)_6_^3−^/Fe(CN)_6_^4−^ under the same conditions (Table 1). In addition, the number of electrons participating in the ER of compound **1a** was determined from the value of the limiting current at the rotating disk electrode (n_e_ RDE), the value of the chronoamperogram current at t = 1 s (n_e_ CA), and the amount of electricity in the interval *t* from 1 to 2 s, in comparison with the amount of electricity passing under the same conditions at the potential of the limiting recovery current of the model compound (n_e_ CC). The results are presented in Table 1, and similar values for **2a** are given for comparison.

The value of the effective number of electrons n_e_ of the first stage of the electroreduction of compound **2a** was equal to 4e, which suggests the reduction of the nitro group to hydroxylamine [20]. Under the same conditions, the value of n_e_ during the electroreduction of compound **1a** is approximately the value 6e. That is, it can be assumed that the intermediate electroreduction products of compound **1a** are electrochemically active at the same potentials, in contrast to the electroreduction products of compound **2a**, which are reduced at more negative potentials.

**Figure 2:**
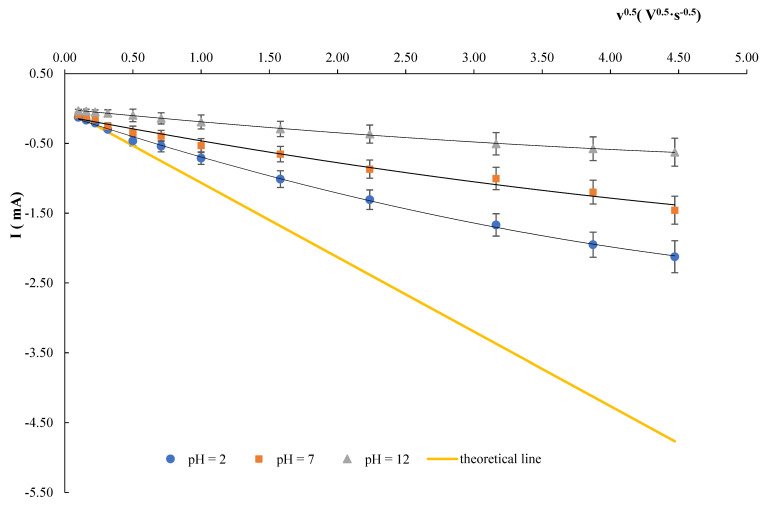
Change in the current of the reduction peak of 5 mM compound **1a** from υ^0.5^ in the BR buffer solution at a pH of 2, 7, and 12 and the theoretically calculated current level according to the Rendles–Shevchik equation for irreversible electrochemical reactions (n = 6e). Potentials were measured relative to Ag/AgCl/KCl_sat_.

The graph of changes in the limiting current ER of compound **1a** (I*_lim_*) at a pH of 2 on voltammograms obtained at different speeds of rotation of the disk electrode (ω) in the coordinates I*_lim_* from ω^0.5^ is linear but does not pass through the origin of the coordinates (Figure S2 in Supplementary Materials). The current of the ER peak in the CV curves versus υ^0.5^ also grows nonlinearly (Figure 2), and the degree of deviation from the straight line depends on the acidity of the medium.

As shown in Figure 2, the value of the peak current of the ER of **1a** deviated from that theoretically calculated by the Rendls–Shevchik equation for irreversible processes [43] at high values of *v* ≥ 0.75 V·s^−1^ in the acidic region. At pH 7, the peak current was close to the theoretically calculated one only at low *v*. The decrease in the peak current for the ER of **1a** in alkaline media (pH > 10) at all values of the potential v is explained [17] by the insufficient protonation rate of the RA due to the low proton concentration. It follows from these data that the electrochemical reaction of the ER of **1a** is controlled by kinetics.

**Figure 3:**
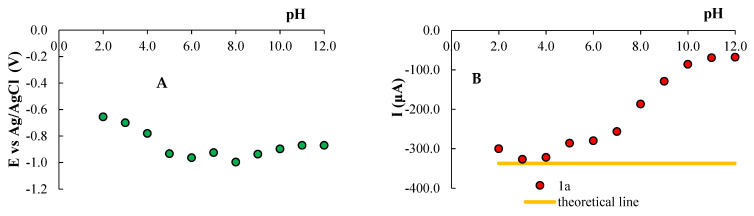
Values of potential (**A**) and current (**B**) of the first peak of the ER 5 mM compound of **1a** with a change in the pH of the buffer solution, *v* = 100 mVs^−1^. Red dots—current calculated by the Rendles–Shevchik equation for irreversible electrochemical reactions involving six electrons. Potentials are measured relative to Ag/AgCl/KCl_sat_.

The reduction current in the CV of compound **1a** (Figure 3B) in an acidic medium, within the measurement errors, had a maximum current value close to the value of the transfer of 6e to the **1a** molecule. At a pH above 4 and in the neutral pH range, the current gradually decreased. At pH values of 10–12, the value of current the ER of compound **1a** practically did not change and was only approximately 20% of the maximum value of the ER current of **1a**. Such a decrease in the current of the ER of **1a** is probably associated with a decrease in the rate of protonation of intermediate anionic species due to the low concentration of protons. However, the electroreduction of compound **1a** remained irreversible under these conditions.

With an increase in the pH of the background electrolyte solution of 3 < pH < 6, the maximum of the reduction peak **1a** shifted toward negative potential values (Figure 3A), with ΔE/ΔpH = 30 mV, and at a pH > 6, it remained almost unchanged.

Preparative electrolysis of compound 1a was carried out in an acidic medium (for electrolysis conditions of 1a, see Supplementary Materials). The obtained data from the electrochemical studies of both NMR and mass spectroscopy (Figures S3 and S4 in Supplementary Materials) suggest that the ER of **1a** in aqueous acidic media proceeds at the nitro group of the compound with the participation of 6 electrons per molecule and leads to the formation of the corresponding heterocyclic amine:

^–^HetNO_2_ NH_3_^+^-R-COOH + 6e + 6H^+^ → HHetNH_2_ + NH_3_^+^-R-COO^−^ +2H_2_O

The end product of electroreduction, 5-Methyl-6-amino-7-oxo-4,7-dihydro-1,2,4-triazolo[1,5-a]pyrimidine, was isolated at a yield of 82% after electrolysis of the solution of **1a** in a BR buffer solution at a pH of 2.

The product was obtained as a precipitate upon cooling the catholyte and was not associated with l-arginine (Figures S3 and S4 in Supplementary Materials).

It is known [19,44,45] that in some cases, when an organic solvent is added to aqueous buffer solutions, the six-electron peak of the nitro group reduction can be divided into two stages. In the CV data of compound **1a,** two stages of electroreduction were observed (5 mM) when 40% DMF was added to the Britton–Robinson buffer solution in the range of 9 > pH > 12, but both stages remained irreversible. In acidic and neutral media, the addition of 40% DMF or more did not lead to a separation of the ER peak of compound **1a** (Figure S5 in Supplementary Materials) These results indicate a high rate of protonation of the formed intermediate particles, which results in the formation of compounds electrochemically active at these potentials.

A comparison of the CV data for compounds **1a** and **1b** (Figure 1) indicated another interesting detail: the currents of the ER of **1a** and **1b** were practically equal, while the ER potentials of these compounds in 0.1 M HNO_3_ differed noticeably by approximately 150 mV. However, it would seem that because both compounds were in an acidic medium, they should be protonated and reduced in the same protonated form. The difference in ER potentials is probably determined by the influence of counterions. The formation of ion pairs of alkali metal ions and small organic cations with anionic particles has been the subject of previous studies [46,47]. They showed that the formation of ion pairs leads to a change in the distribution of electron density in the radical anion (RA), which affects the reduction potential of compounds. The difference in the ER potentials of compounds **1a** and **1b** is probably related to the effect of Na^+^ ions; 5 mM Na^+^ is added to the solution together with **1b**, and in the case of the ER of **1a**, the bulky amino acid cation should have little effect on the electron density distribution in RA **1a**. In a BR solution at a pH of 2, a slight decrease in the peak current and a slight shift in the potential of the ER of **1a** to the positive region (−0.63 V) compared with the HNO_3_ solution are possibly related to the presence of Na^+^ ions.

### 3.2. Electroreduction of Compound **1a** in Aprotic Media

**Figure 4:**
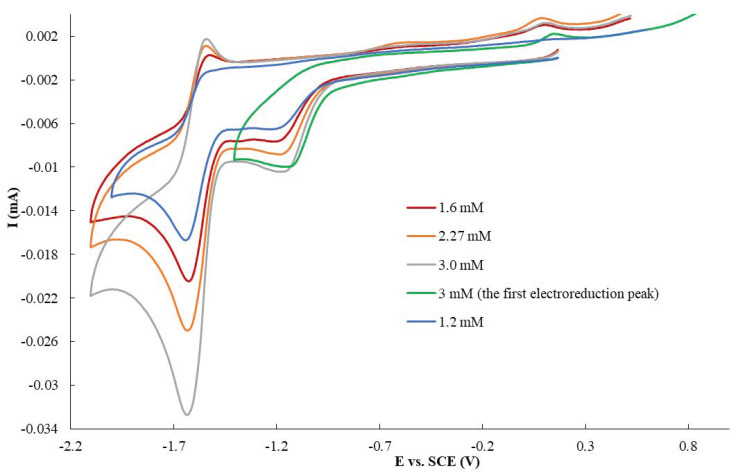
CVs obtained in the presence of compound **1a** at various concentrations, 1.2, 1.6, 2.3, and 3.0 mM, in a DMF solution (0.1 M Bu_4_NClO_4_). *v* = 100 mVs^−1^.

In an aprotic medium, anionic intermediate particles formed during reduction are protonated more slowly than they are in an aqueous medium. Therefore, a study of **1a** in DMF and acetonitrile was carried out with the aim of registering intermediate electroreduction particles. The electrochemical behavior of **1a** in aprotic solvents was found to be similar. In the CV results **1a** in DMF, two ER peaks were observed (Figure 4). The first of them was an irreversible flat ER peak at −1.22 V, with the next stage at −1.63 V.

**Figure 5:**
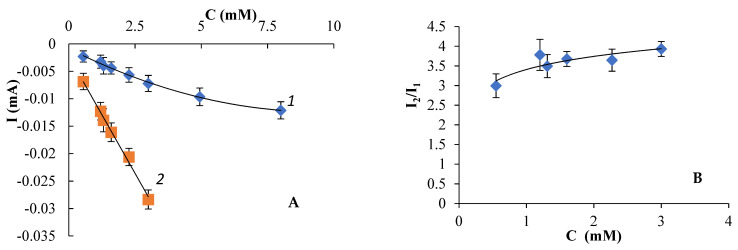
(**A**) The values of the current on the CVA of compound **1a** at the potentials of the first (1) and second (2) stages versus concentration. (**B**) Change in the ratio of currents at the potentials of the second and first stages (I_2_/I_1_) from the concentration of **1a**. *v* = 100 mVs^−1^.

According to the CV and CA data, the current value of the first stage of the ER of **1a** was noticeably lower than the current of the peak of the one-electron oxidation of ferrocene under the same conditions and varied nonlinearly in the concentration range of 0.6 to 8 mM (Figure 5A). In the range of working concentrations of 1–3 mM, the current of the first peak is was approximately half the current of a one-electron transfer.

**Figure 6:**
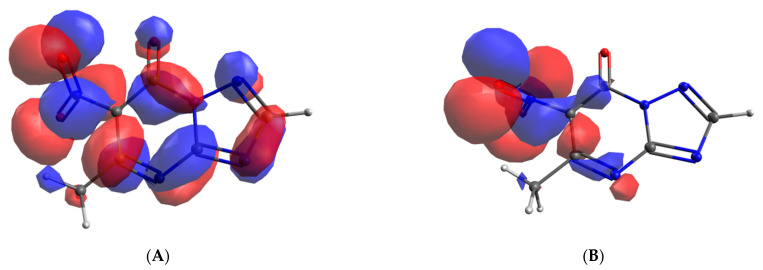
Orbital plot of the LUMO (**A**) of heterocyclic anion **1a** and the SOMO (**B**) and its DAR (from PBE0/D3BJ/def2- TZVPP calculations).

As shown by the results of the CV of l-arginine hydrochloride, neither the l-arginine cation nor its anion obtained by adding alkali were not reduced under these conditions. No paramagnetic activity was recorded in l-arginine solutions when the potential was applied under CV conditions. This indicates that, most likely, an electron is transferred to the heterocyclic part of salt **1a**. According to the results of quantum-chemical calculations of the energies of the lowest vacant molecular orbital of the heterocyclic anion (LUMO) and the energy of the single-occupied molecular orbital (SOMO) of the dianion radical (DAR) formed during its ER (Figure 6), the most likely explanation that the transfer of electrons to the nitro group and a double bond of a six-membered ring.

Since **1a** is a salt formed by a heterocyclic anion and an amino acid, it can be assumed that the transfer of the first electron at the potentials of the first stage is accompanied by rapid protonation of the formed ER product by the initial depolarizer—so-called self-protonation [48,49]. The amino acid l-arginine, which is part of compound **1a**, is a good proton donor. The pKa value of the first stage in the aqueous medium is 2.17 [50]. The pKa value of compound **1a** in water was determined potentiometrically to be 3.45. Therefore, in an aprotic medium, **1a** can act as a proton donor.

**Figure 7:**
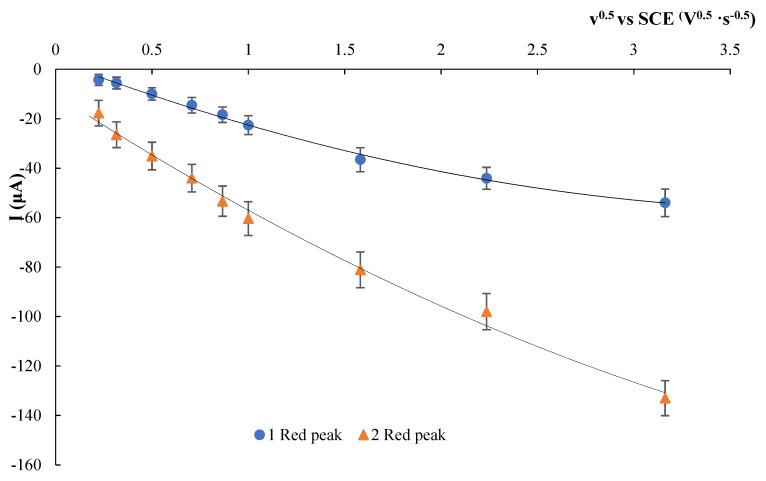
Change in the current of the reduction peaks for 3 mM compound **1a** from *v*^0.5^ in DMF (0.1 M Bu_4_NBF_4_).

The dependence of the current of the first peak I_1_ of the reduction of **1a** on *v*^0.5^ in an aprotic medium (Figure 7) is linear and passes through the origin only in the region of low rates of potential application <2 Vs^−1^.

The obtained data allow us to assume that at the potentials of the first stage, E_1_, the most rapid reaction is self-protonation leading to the formation of an electrochemically inactive heterocyclic anion Het^−^ at E_1_:

Het^–^NH_3_^+^-R-COOH + e → [Het ^–^NH_3_^+^-R-COOH]^−^ *E*_1_

[Het ^–^NH_3_^+^-R-COOH]^−^ + Het ^–^NH_3_^+^-R-COOH → [Het ^–^NH_3_^+^-R-COOH]H^·^ + Het ^−^ + NH_3_^+^-R-COO^−^

Proton transfer can probably proceed not only intermolecularly, but also intramolecularly, as well as through several reaction centers. As a result, the formation of radicals with different positions of the spin density of the unpaired electron and, consequently, different reactivity is possible (Supplementary Materials Scheme S1).

The second stage of the electroreduction of compound **1a** is quasi-reversible. On the reverse curve after the second peak in the concentration range of 1.6–3.0 mM, an anodic peak was recorded at E = −1.54 V and ΔEp was approximately 90 mV. The value of the current at the potentials of the second stage of the ER I_2_ increased with increasing concentration (Figure 5A), but the linear plot of the dependence of I_2_ in the C_1a_ 0.6–3.0 mM interval does not pass through the origin. With an increase in the concentration of 1a, the ratio of the currents at the potentials of the second I_2_ and the first I_1_ stages I_2_/I_1_ also increased (Figure 5B), which suggests reduction at the potentials of the second stage of the second-order chemical reaction product. The potentials of the second peak, mainly the heterocyclic anion formed at the potentials of the first stage of the ER of **1a,** are probably reduced.

Het ^–^ + e → [Het] = *E*_2_

The oxidation peak of the dianion radical (DAR) on the reverse at a potential of −1.54 V was approximately half of the second cathodic peak at *v* = 0.1 Vs^−1^ (Figure 4), and with an increase in *v* to 5 Vs^−1^, the value of I_a_/I_c_ increased to 0.74.

The peak current of the second stage of electroreduction of compound **1a** from *v*^0.5^ in an aprotic medium changed nonlinearly (Figure 7). An increase in the v led to a relative decrease in the currents of both cathodic peaks. Moreover, the I_2_/I_1_ ratio at a high v (above 2 Vs^−1^) remained constant and approximately equal to 2, while at *v* = 50 mV s^−1^, it exceeded 4. It can be assumed that the protonation reaction of DAR (with the initial compound and/or donor impurities in the solution) has time to proceed at low values *v*, which leads to an increase in the second cathodic peak and a decrease in the anodic peak at −1.54 V in the CV of compound **1a.**

**Figure 8:**
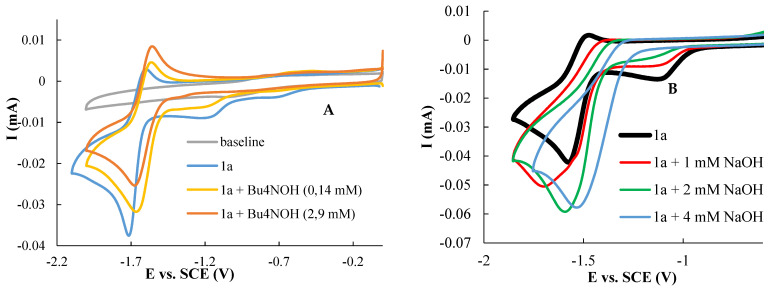
(**A**) CVs obtained in the presence of compound **1a** (3 mM) in a solution of DMF (0.1 M Bu_4_NBF_4_) *v* = 0.1 Vs^−1^ with the addition of an aqueous solution of Bu_4_NOH. (**B**) CVs obtained in the presence of compound **1a** (3.3 mM) in a DMF solution (0.1 M Bu_4_NClO_4_) *v* = 0.1 Vs^−1^ with the addition of an aqueous solution of NaOH.

The gradual addition of Bu_4_NOH alkali caused a decrease and complete disappearance of the current of the first stage (Figure 8A) at an equivalent alkali content, while the current value of the second stage remained practically unchanged.

It is interesting to note that the anodic peak of −1.54V increased under these conditions, and the I_a_/I_c_ value was 0.7 when the Bu_4_NOH content was two times higher than the **1a** concentration. This result can be considered a confirmation of the assumption that the DAR formed at the potentials of the second stage of the ER of compound **1a** disappears as a result of the protonation reaction. An increase in the rate of potential deposition or a decrease in the content of proton donors due to the addition of alkali makes it possible to register a higher current of its oxidation at a potential of −1.54 V:

Het ^–^NH_3_^+^-R-COOH + OH^−^→ Het ^–^ + NH_3_^+^-R-COO^−^ +H_2_O

Het ^–^ + e → [Het] =

The formation of dianion radicals was also shown for a number of 1,2,4-nitrotriazoles [51].

It is important to note that the addition of an aqueous NaOH solution to a solution of **1a** had a different effect on the cathodic peaks of the CVs of the compound (Figure 8B). The addition of NaOH to a solution of **1a** in DMF also led to the disappearance of the first stage of the ER. The second stage became irreversible, while the potential of the ER shifted to more positive potentials, and the current increased. Apparently, these changes can be associated with the formation of ion pairs between the heterocyclic anion **1a** and Na^+^ ions, in which the electron density at the reaction center increases under the influence of the cation, and the protonation of DAR proceeds at a higher rate. When Bu_4_NOH was added to the solution instead of NaOH (Figure 8A), ion pairs with the heterocyclic anion were not formed since the charge density in the bulk cation Bu_4_N^+^ is significantly lower than that in the Na^+^ ion. As a result, the reversible ER of the **1a** anion was observed on the CVs.

**Figure 9:**
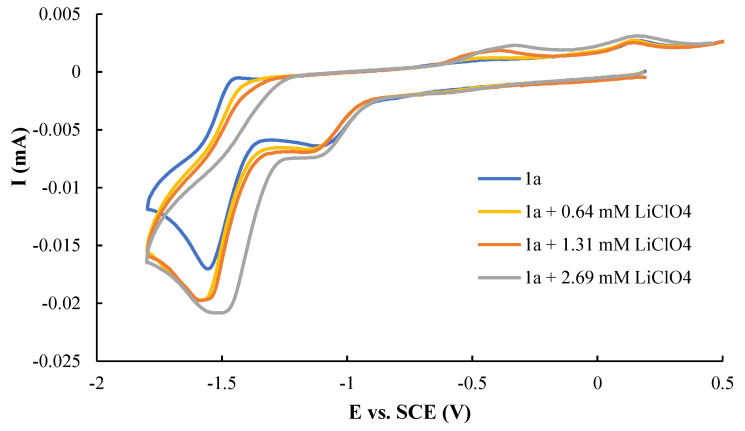
CVs of a 1.3 mM solution of **1a** in the presence of LiClO_4_, *v* = 0.1 Vs^−1^.

Figure 9 shows the cyclic voltammograms of the solution of **1a** in the presence of added LiClO_4_. These were made in order to exclude the effect of the water added with alkali to the DMF solution and to study the effect of a cation even smaller than Na^+^.

By comparing the influences of LiClO_4_ and NaOH, it can be noted that the presence of LiClO_4_, in contrast to that of NaOH, does not affect the first stage of the ER of **1a**, but the second stage of ER in the presence of both compounds becomes irreversible and the reduction current increases, probably due to the protonation of the resulting products. In addition, in both cases, oxidation peaks appear on the anodic reversal curve at close potentials in the range of −0.5 to −0.3V. It is possible that in the presence of alkali metals, not only does the rate of protonation of **1a** dianion radicals change, but the regioselectivity of the chemical reaction also changes. It should be emphasized that the ESR signal could not be detected in the presence of Li^+^ ions when LiOH was added to a solution of **1a** in DMF.

### 3.3. Study of the Products of Electroreduction of Compound **1a** by ESR Spectroscopy

The electron density of the lowest free molecular orbital (LUMO) of the heterocyclic anion **1a** was theoretically calculated to determine the possible reduction centers (Figure 6). From these calculations, two possible pathways for the formation of radicals were suggested. The first, which is the most probable, is the reduction of the nitro group with the formation of a nitroxyl radical, and the second is the formation of a radical on the nitrogen at the 4 position of the six-membered ring of the heterocycle.

Preliminary reduction of **1a** was carried out at a peak potential of E = −1.7 V in a DMSO solution in the presence of a TMTH spin probe to detect the formation of radical products during the ER of **1a**. The obtained experimental data indicate the appearance of radicals in the solution of the electrochemical cell during electrolysis. A significant increase in the number of paramagnetic centers in the solution of the electrochemical cell during the entire electrolysis process for **1a** (Figure S6 in Supplementary Materials) was observed. In addition, a linear relationship between the number of spins and the time spent on the electroreduction of the products of **1a** was observed (Figure S6 tab in Supplementary Materials).

The high reactivity of the anionic particles formed during the ER of **1a**, primarily in the protonation reactions, makes it difficult to obtain high-intensity ESR spectra. Therefore, the ER of **1a** c was carried out in an SEESR experiment in the presence of Bu_4_NOH.

**Figure 10:**
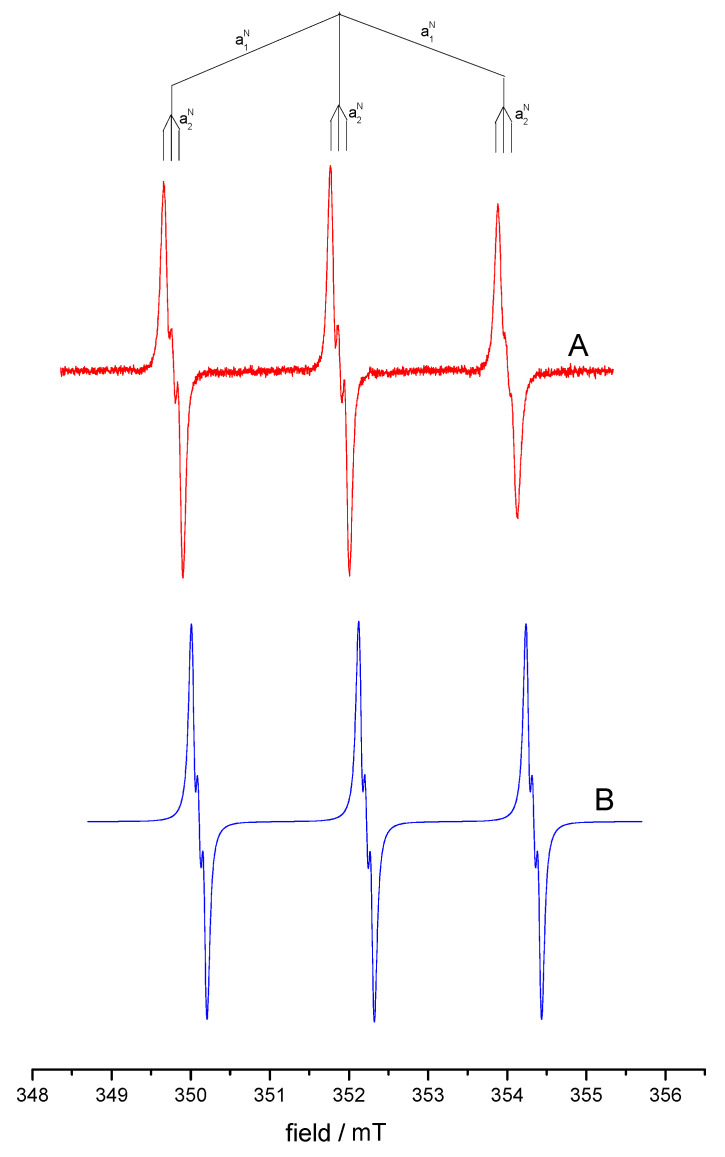
ESR spectra of the radical dianion: experimental (**A**) (room temperature, DMF/Bu_4_NClO_4_/Bu_4_NOH) and simulated isotropic spectrum (**B**) triplet of triplets (g_i_ = 2.0059, $a_{1}^{N}$ (spin = 1) = 2.12 mT, $a_{2}^{N}$ (spin = 1) = 0.7 mT, LW = 0.065 mT, L/G = 0.1).

The SEESR experiment was used for the detection of paramagnetic species during the reduction of **1a**. Figure 10 gives examples of the experimental and simulated spectra of the radical anion generation recorded at a potential of −2 V in a two-electrode Pt/Pt electrochemical cell placed in the resonator of an ESR spectrometer. The half-life of the detected species was less than 0.01 s.

The isotropic spectrum of the radical dianion recorded at room temperature was a triplet of triplets—three equidistant lines by splitting on the nitrogen (spin = 1) of the NO_2_ group ($a_{1}^{N}$ = 2.12 mT), with each of them split by a small constant ($a_{2}^{N}$ = 0.7 mT) on the nitrogen (spin = 1) in position 4 of the heterocycle.

Calculated using the DFT level of theory (PBE0/def2-TZVPP), the hyperfine coupling constants for the dianion radical of 1A—g_i_ = 2.0057, $a_{1}^{N}$ (NO_2_ group) = 2.28 mT, and $a_{2}^{N}$ (N in the 4 position of the heterocycle) = 0.44 mT—were in agreement with the experiment.

The ESR spectra of the adducts of the TEMPO spin trap with the reduction product **1a** are additional evidence of the structure of the radicals formed during the ER of **1a** (Figure S7 in the Supplementary Materials). The obtained ESR spectrum can be described as a superposition of the ESR spectra of two adducts; in one adduct, the center of the interaction of the spin trap with the supposed radical is the nitro group, and in the second, the center is the nitrogen atom in position 4 of the six-membered heterocycle.

## 4. Conclusions

The results of this study of the electrochemical behavior of compound **1a** (5-methyl-6-nitro-7-oxo-4,7-dihydro-1,2,4-triazolo[1,5-a]pyrimidinide l-arginine monohydrate) in an acidic aqueous medium showed that the electroreduction of the nitro group of this compound proceeds irreversibly in one six-electron step with the formation of the corresponding heterocyclic amine. The ER of **1a** proceeds at more negative potentials in comparison with that of the previously studied **2a** in both aqueous and aprotic media. Compound **1a** is reduced in two stages in aprotic media. The transfer of the first electron is accompanied by the competition of protonation reactions with the participation of the starting compound **1a**. At the potentials of the second stage, the heterocyclic anion **1a** is reversibly reduced to form the dianion radical. The ESR spectrum of this particle was recorded upon the ER of compound **1a** in the presence of Bu_4_NOH.

The effect of Na^+^ and Li^+^ ions added to the DMF solution was considered. In the presence of these ions, not only does the rate of protonation of the dianion radicals of the investigated compound change, but the regioselectivity of this reaction also changes. The results of ESR spectroscopy make it possible to assume the formation of both a nitroxyl radical and a radical with spin density localization on nitrogen in the 4 position of the six-membered ring during the ER of **1a**. The obtained ESR spectrum of adducts of radical particles with a TEMPO spin trap can be described as a superposition of the ESR spectra of these radicals.

## Figures and Tables

**Table 1:** The values of the observed number of electrons n_e_ involved in the electroreduction of compound **1a** (C = 5 mM) in an aqueous buffer solution of BR at a pH of 2, determined by CV, CA, CC, and RDE methods.

Compound	n_e_	n_e_ (CA)	n_e_ (CC)	n_e_ (RDE)
**1a**	5.63	5.72	5.86	4.59
**K_3_Fe(CN)_6_**	1	1.02	1	0.96
**2a** [20]	3.25	4.08	4.17	3.68

## Data Availability

The data presented in this study are available on request from the corresponding author.

## Supplementary Materials

The following are available online. Figure S1: CVs obtained in BR solution and compound **1a** before background discharge. Figure S2: The dependence of the limiting current of the electroreduction of compound **1a** (C = 5·10^−3^ mM) on ω^0.5^ in a BR solution with pH = 2. Figure S3: NMR spectrum of the product after electrolysis of **1a**. Figure S4: Mass spectra of products after electrolysis of **1a**. Electrolysis of compound **1a** in an acidic medium. Figure S5: CVs of **1a** in a 60% Britton–Robinson buffer with 40% DMF. Sweep scan 50 mVs^−1^: 1—pH = 2; 2—pH = 9; 3—pH = 12. Scheme S1: Possible further ways of reducing **1a**. Figure S6: ESR spectra of the products of the reduction reaction of compound **1a** with the TMTH probe and the relative number of paramagnetic centers after 0, 1, 5, 10, and 15 min of electroreduction at −1.7 V. Figure S7: ESR spectra of TMPO spin trap adducts with a reduction product **1a**.

## Sample Availability

Samples of the compounds 5-Methyl-6-nitro-7-oxo-4,7-dihydro-1,2,4-triazolo[1,5-a]pyrimidinide l-arginine monohydrate (**1a**, Triazid), sodium salt of 5-methyl-6-nitro-7-oxo-4,7-dihydro-1,2,4-triazolo[1,5-a]pyrimidine (1b), sodium salt of 2-methylthio-6-nitro-7-oxo-1,2,4-triazolo-[5,1-c][1,2,4]triazin, dihydrate (**2a**, Triazavirin^®^), and 2-methylthio-6-nitro-7-oxo-1,2,4-triazolo-[5,1-c][1,2,4]triazin (**2b**) are available from the authors.
