# Supplementary material for: Redox Conversions of 5-Methyl-6-nitro-7-oxo-4,7-dihydro-1,2,4triazolo[1,5-a]pyrimidinide L-Arginine Monohydrate as a Promising Antiviral Drug
Source: Molecules. 2021 Aug 22;26(16):5087. doi: 10.3390/molecules26165087 (PMC8400642; doi:10.3390/molecules26165087)
Supplement: Supplementary file 1 [file molecules-26-05087-s001.zip › molecules-1321304-supplementary.pdf]

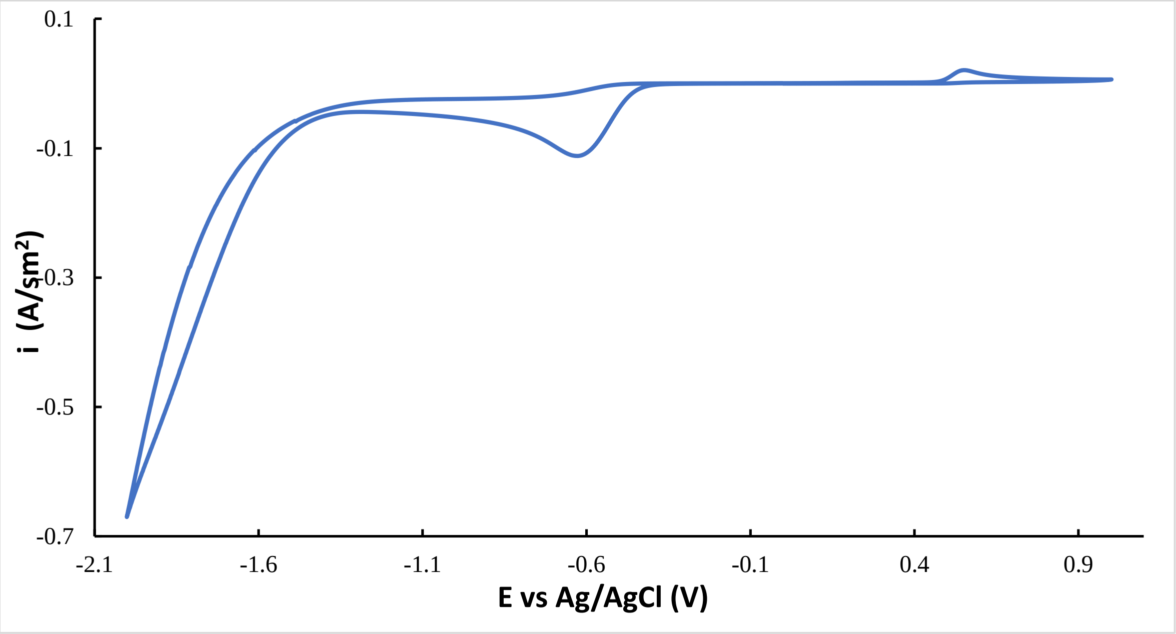

Figure S1. CV obtained in BR solution and compound **1a** before background discharge.

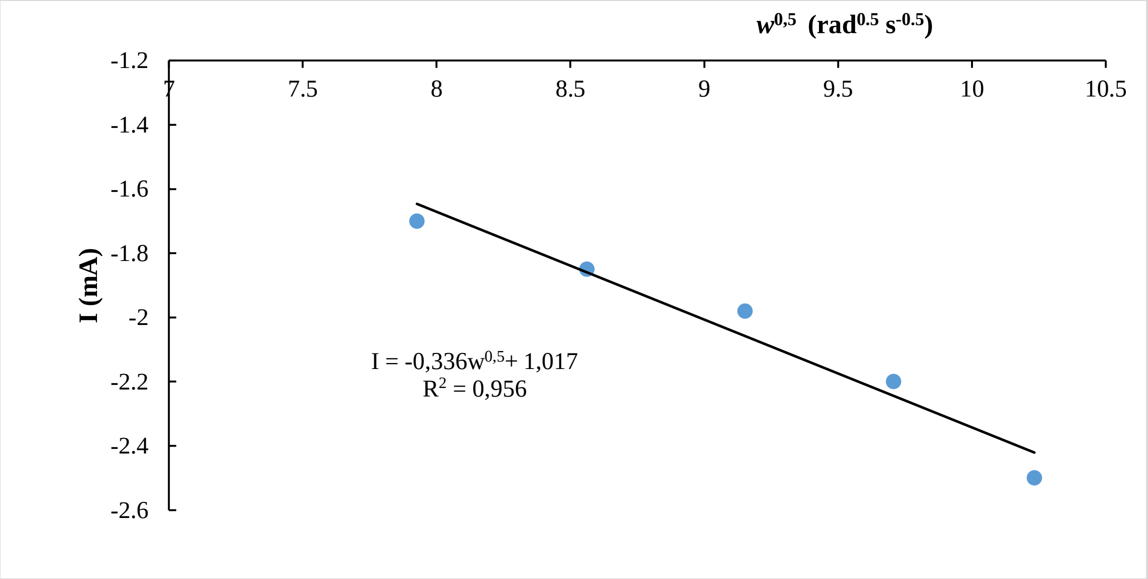

Figure S2. The dependence of the limiting current of electroreduction of compound **1a** ($C=5\cdot10^{-3}$ mM) on $\omega^{0.5}$ in a BR solution pH = 2.

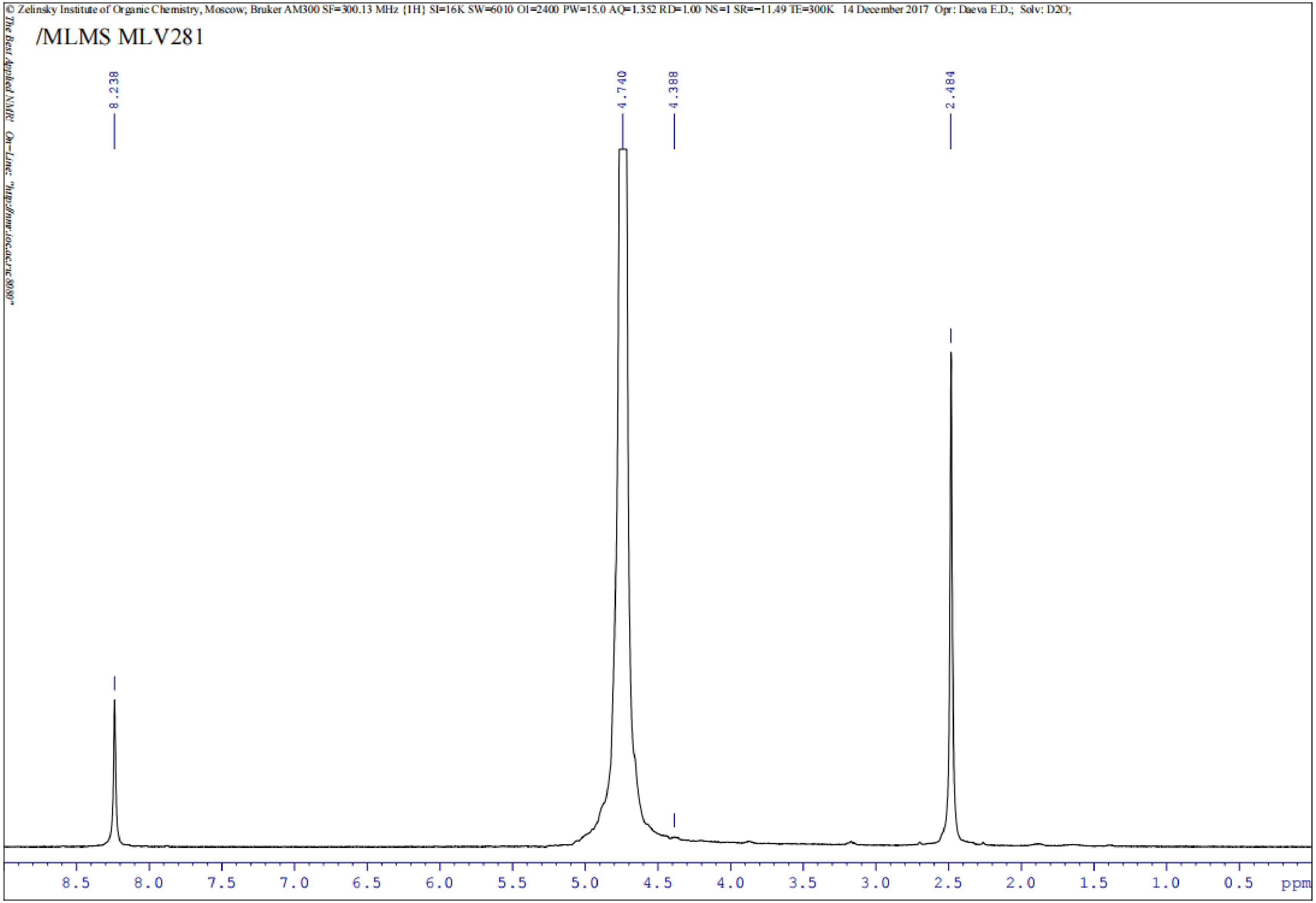

Figure S3. NMR spectrum of the product after electrolysis of **1a**.

File : C:\MSDCHEM\1\DATA\BA0400P.D
Operator :
Acquired : 14 Dec 17 14:21 using AcqMethod DIP_1.M
Instrument : INCOS50
Sample Name: MIKH-3
Misc Info :
Vial Number: 1

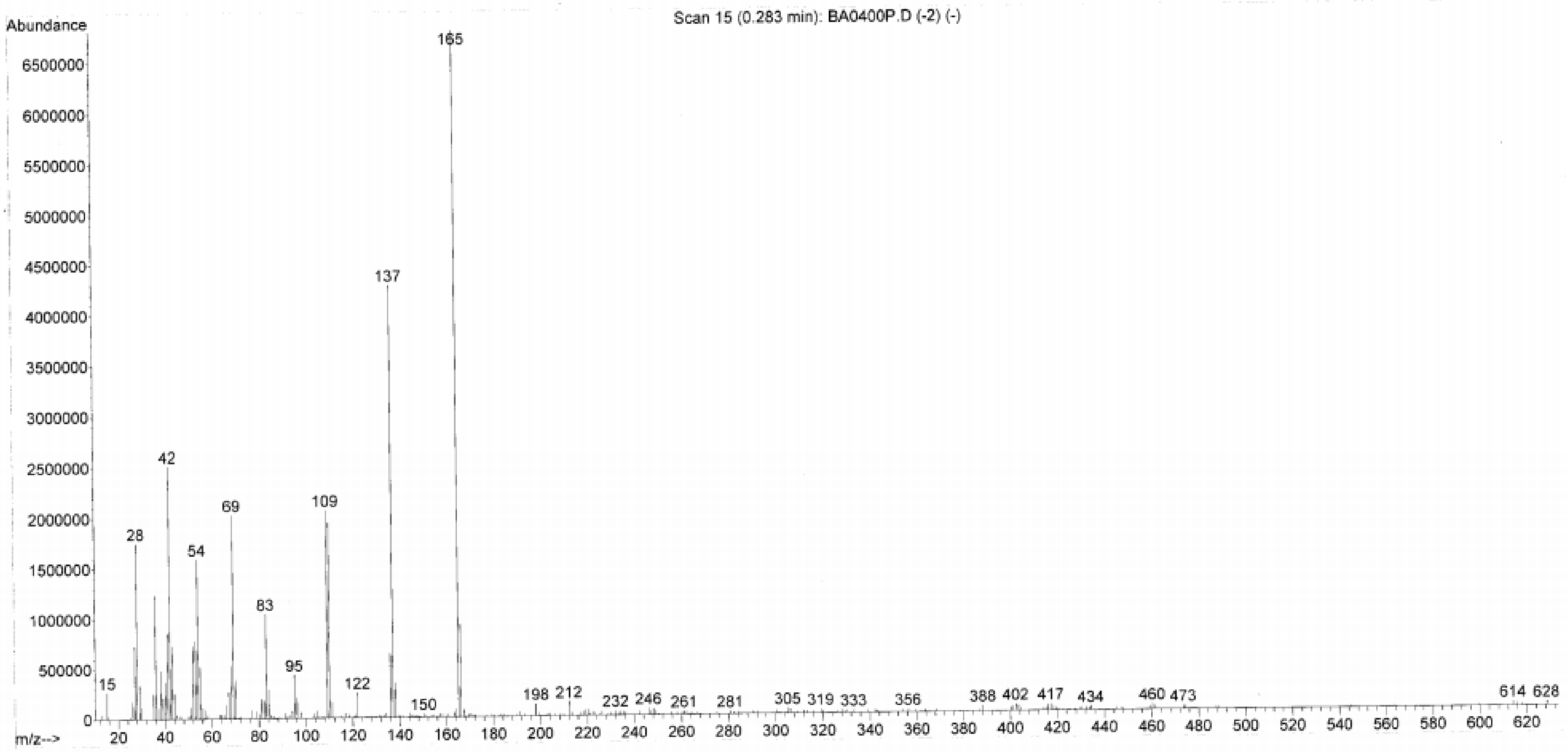

Figure S4. Mass spectrum of products after electrolysis of **1a**.

*Electrolysis of compound **1a** in an acidic medium*

The electrolysis of compound 1a was carried out in an open-type two-electrode cell with anode and cathode compartments separated by a cation-exchange membrane. Catholyte - buffer solution BR, pH 1.8 - 2.2, anolyte - 15% H_2SO_4. A glassy carbon glass with an insulating support was used as the cathode; the working surface area was ≈ 43.2 cm^2. The anode is a graphite rod with a geometric area of the working surface of ≈ 85.3 cm^2. The catholyte was stirred with a magnetic stirrer and purged with argon. The electrolysis was carried out in a galvanostatic mode at a current density of 5 mA / cm^2. The concentration of **1a** was monitored by electroanalytical methods. 6 F / mol was passed through the solution, 85% conversion. The white precipitate that formed during electrolysis and as a result of cooling the catholyte was washed with dionized water, dissolved in acetone, and dried under vacuum. Isolated 5-Methyl-6-amino-7-oxo-1,2,4-triazolo [1,5-a] pyrimidine, 0.07g (82%).

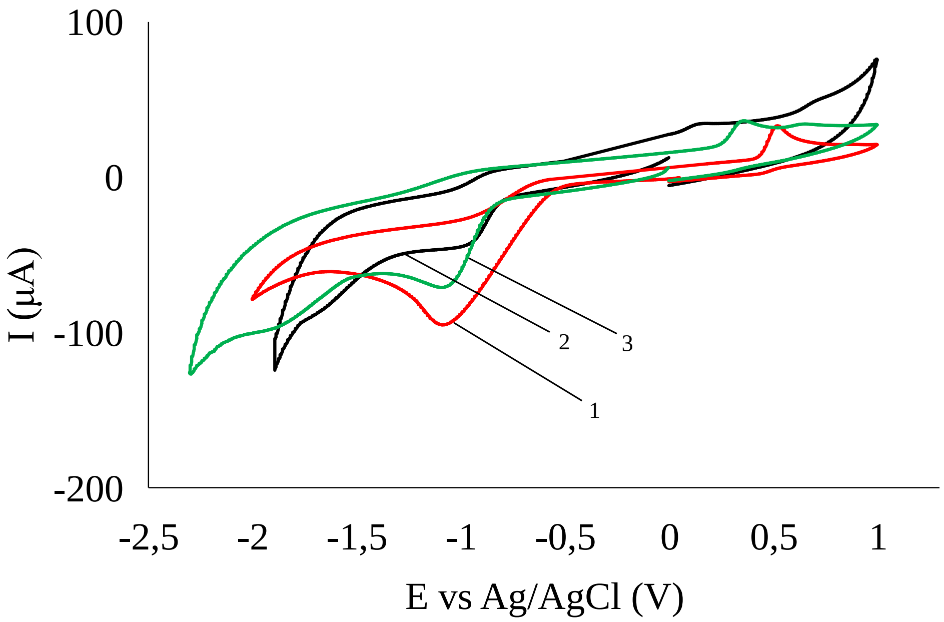

Figure S5. CVs of **1a** in a 60% Britton-Robinson buffer with 40% DMF. Sweep scan 50mVs^{-1}: 1 – pH=2; 2 – pH=9; 3 – pH=12.

Scheme 1S. Possible further ways of reduction **1a**.

$[Het^{-}NH_3^{+}\text{-R-COOH}]H^{\cdot} \rightarrow [Het\,H_2]^{\cdot} + NH_3^{+}\text{-R-COO}^{-}$

$[Het^{-}NH_3^{+}\text{-R-COOH}]H^{\cdot} \rightarrow [Het\,H]^{\cdot-} + NH_3^{+}\text{-R-COOH}$

$[HetH_2]^{\cdot} + e \rightarrow HetH_2^{-}$ (1)

$[Het\,H]^{\cdot} + e \rightarrow HetH^{-}$ (2)

$[Het\,H]^{\cdot-} + Het^{-}NH_3^{+}\text{-R-COOH} \rightarrow {}^{-}[Het\,H]\text{-}[Het\,H]^{\cdot} + NH_3^{+}\text{-R-COO}^{-}$

${}^{-}[Het\,H]\text{-}[Het\,H]^{\cdot} + e \rightarrow {}^{-}[Het\,H]\text{-}[Het\,H]^{-}$ (3)

${}^{-}[Het\,H]\text{-}[Het\,H]^{-} + Het^{-}NH_3^{+}\text{-R-COOH} \rightarrow {}^{-}[Het\,H]\text{-}[Het\,H]H + Het^{-} + NH_3^{+}\text{-R-COO}^{-}$

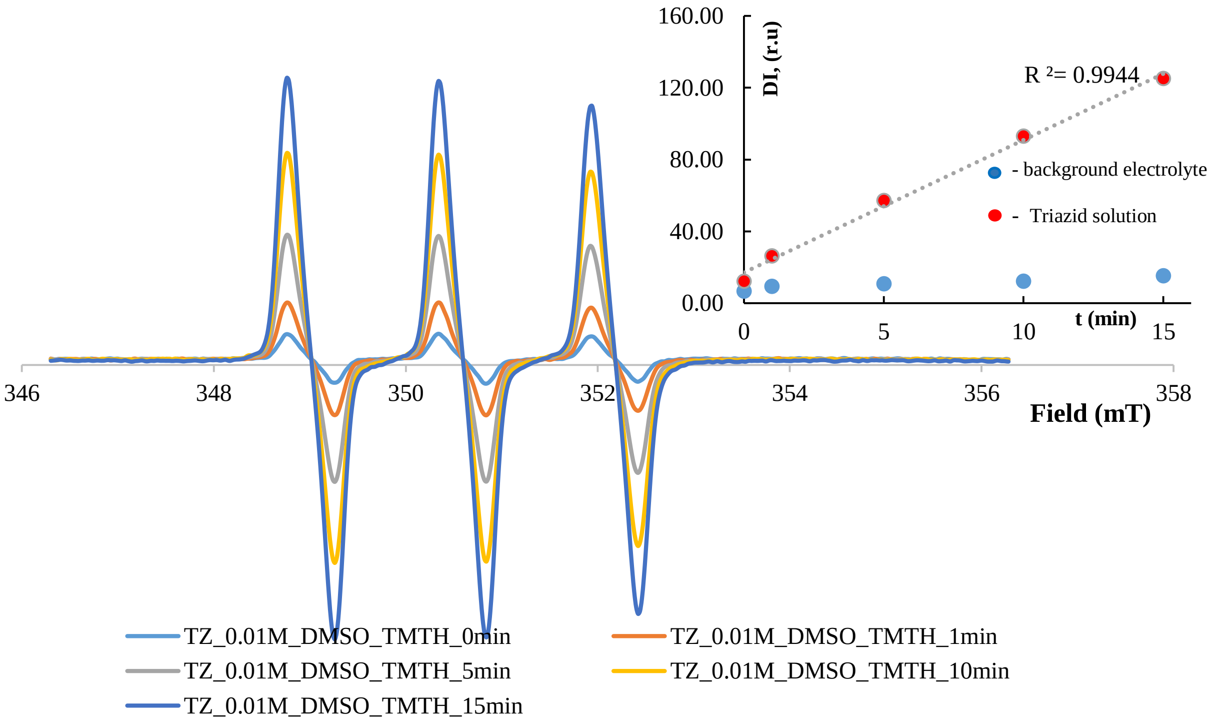

Figure S6. ESR spectra of the products of the reduction reaction of compound **1a** with the TMTH probe and the relative number of paramagnetic centers after 0,1, 5,10,15 min of electroreduction at -1.7V.

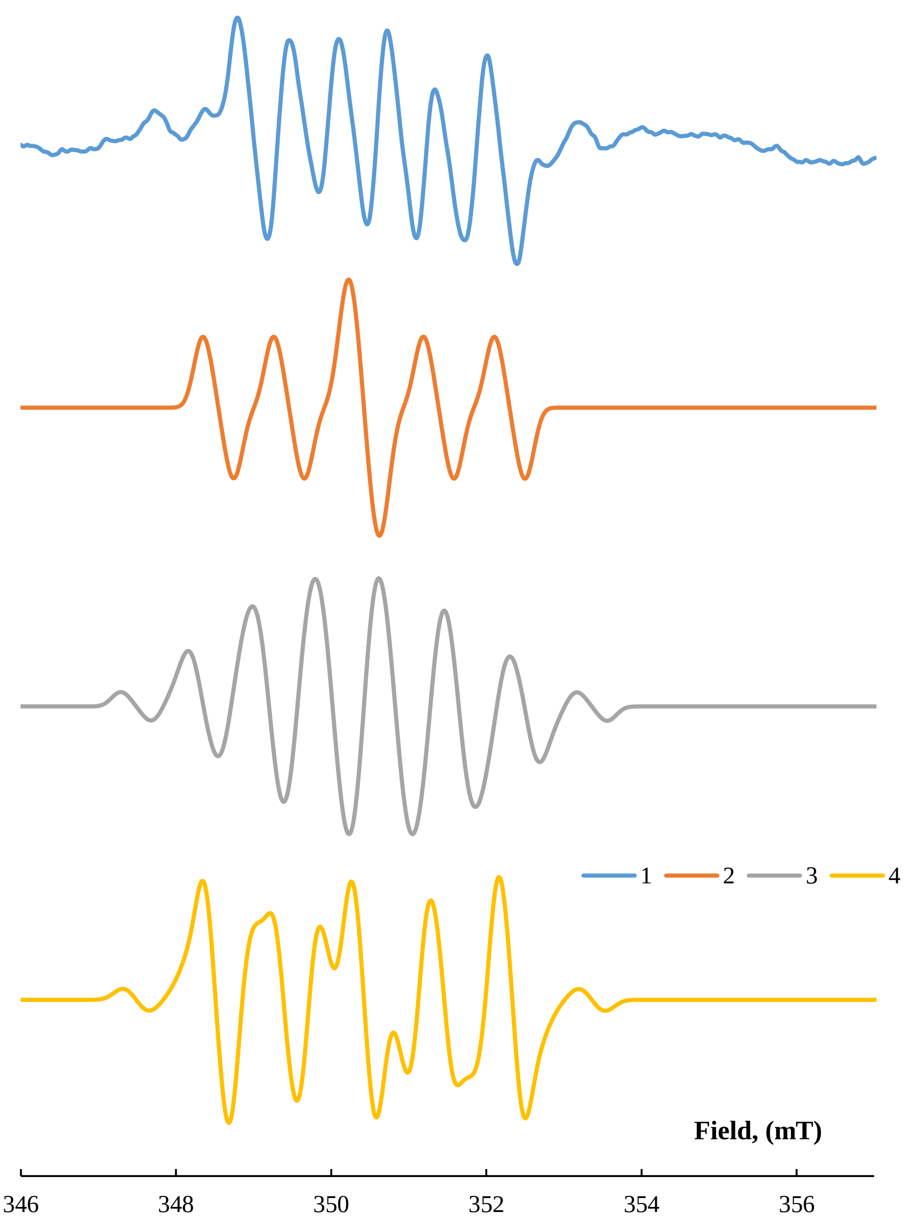

Figure S7 - ESR spectra of TEMPO spin trap adducts with a reduction product **1a**: 1) experimental spectrum, 2) the calculated spectrum of the trap adduct after interaction with the nitro group of the radical, 3) the calculated spectrum of the trap adduct after the interaction with the nitrogen atom in the 4 position in the six-membered heterocycle, 4) superposition of 2 and 3 spectra in the ratio of radicals 1: 1. The calculated spectra are modeled with LW = 0.2mT, L / G = 0.1. The calculated constants CTB, g_{iso}, are loaded from the * .prop files of the corresponding Orca calculations. Simulation error between 1 and 4 (Root mean square deviation) r_{msd} = 2.12%.
